# Enhancing the collective, protecting the personal: the valuable role of composite narratives in medical education research

**DOI:** 10.1007/s40037-022-00723-x

**Published:** 2022-08-11

**Authors:** Zoë McElhinney, Catherine Kennedy

**Affiliations:** 1 General Practice Undergraduate Education, School of Medicine, University of Dundee, Dundee, UK; 2 Centre for Medical Education, School of Medicine, University of Dundee, Dundee, UK

**Keywords:** Composite narrative, Qualitative research methodology, Medical education

## Abstract

Narrative research approaches provide the opportunity for constructing a detailed understanding of lived experiences relevant to medical education, in areas such as illness narratives, explorations of doctor-patient relationships, and the development of professional identities in students and educators. The benefits of the depth of data gathered in narrative research are, however, counterbalanced by possible weaknesses relating to a focus on individual cases and the risk of identification of participants where subjects are sensitive or unique. To address these concerns, researchers from a variety of social science disciplines, carrying out research employing a range of methodological approaches, have begun to use ‘composite narratives’ in which the commonalities in the experiences of research participants are combined to create joint narrative or narratives which illustrate participants’ shared experiences. Composite narratives have been used both as a component of the methodological approach and as a method of presenting the results of research in a variety of methodologies. This *A Qualitative Space *paper explores the role, strengths, and weaknesses of narrative research, before outlining the ways in which composite narrative has been defined within existing research. Distinctions between the various approaches to creating composite narratives are discussed, highlighting the differences in the types of data utilised, and the approaches taken to data analysis and presentation. A key distinction is identified between the use of composite narratives as part of an integrated methodology and as an approach to the presentation of data. Finally, issues relating to trustworthiness, reflexivity, and implications for researchers are considered.

## A Qualitative Space

A Qualitative Space highlights research approaches that push readers and scholars deeper into qualitative methods and methodologies. Contributors to *A Qualitative Space *may: advance new ideas about qualitative methodologies, methods, and/or techniques; debate current and historical trends in qualitative research; craft and share nuanced reflections on how data collection methods should be revised or modified; reflect on the epistemological bases of qualitative research; or argue that some qualitative practices should end. Share your thoughts on Twitter using the hashtag: #aqualspace

## Introduction

The use of narratives in medical education is well established. Case histories and patient narratives are frequently used in clinical teaching and reflective educational practices such as reflective portfolios and Balint groups [1]. Narrative-based medicine, as advocated by Charon [2], Launer [3], and Greenhalgh and Hurwitz [4] emphasises the importance of narratives in understanding the experience of illness, including the patient’s perspective and context, and in developing the doctor-patient relationship.

Distinct from narrative-based medicine is narrative research, a group of qualitative methods that use the storied accounts of participants to explore the research question [5]. Narratives can be used both as data and as a means of presenting findings [6]. Narrative research can include research that takes a narrative methodological approach, as well as research that employs narratives as a method of data collection or data presentation within another methodological approach, such as phenomenology or ethnography. Although well-established within the social sciences, narrative methods are not as commonly used in medical education research. There are some examples of their use in research exploring the experience of being a doctor, including the development of professional identity [7–9].

In social science research there has been an increase in the use of composite narratives to present research findings. Whilst this has, to date, been a little used method in medical education research, more recently composite narratives have been used in research exploring the experiences of academic GPs [8], and of doctors in Scotland [10] and Ireland [11] during the COVID pandemic.

In this paper we explore composite narratives. Firstly, we examine the context in which this method has arisen, considering narrative research more broadly. We then focus on composite narratives including the various definitions, approaches to production and uses, and opportunities for their use in medical education research.

## Narratives in research

‘Narrative’ as a term has a multitude of meanings and is used differentially between disciplines [5]. It can range in form from the close scrutiny of units of discourse, as within Labovian analysis [12], to the composition of life stories from a range of sources within life history and anthropology [5]. Narrative inquiry is perceived both as a methodology in itself [13] and utilised with a variety of interpretive research approaches that centre the lived experience of the subject such as phenomenology and ethnography [14]. Alternatively, narratives, in the form of written or visual artefacts, can be used as a method of data collection [15, 16]. Polkinghorne [14, p. 12] draws a distinction between ‘the paradigmatic analysis of narrative data’ through which narratives are analysed in relation to themes and categories, and ‘narrative analysis’ in which researchers gather disparate data relating to events and experiences and synthesise them into a story. Bleakley [6, p. 535] refers to this difference (using narrative methods of data collection versus presenting data in a storied form) as being between the use of ‘stories as data’ rather than ‘data as stories’.

The distinctiveness of narrative inquiry relates to its purpose to convey the narrative ‘sense making’ of an experience individuals engage in through the telling of their story. In this way, stories can act as ‘a portal through which their experience of the world is interpreted and made personally meaningful’ [17, p. 375]. Narrative research is also concerned with context, and how a narrative ‘connects events into a sequence that is consequential for later action and the meanings the speaker wants the listeners to take away from the story’ [5, p. 3]. Narrative research typically involves a small number of cases studied in depth, and is valuable when access to detailed stories can help to understand a phenomenon [15]. Exploring the sequence of events, whether through longitudinal data collection or reflection on past experiences, allows an exploration of the temporal sequence, of how stories unfold. The situated nature of narratives, which often focus on the individual, can provide authenticity, texture, and emotional connection [5, 18]. This focus on the individual can highlight both the unique and the particular of the individual’s experience and situate the experience within its wider socio-cultural context. In medical education research, Clandinin et al. [19] used narrative reflective practice in analysing an in-depth case of a single medical student to explore the development of professional identity, whilst Gordon et al. [7] utilised longitudinal narrative inquiry to explore the liminal phases in professional identity formation in the transition from trainee to trained doctor.

Researchers have, however, highlighted several challenges associated with conducting narrative research. These include the difficulties of interpreting the data and conflicts over the ownership, presentation, or interpretations of the story(ies) [20] which can raise ethical challenges for the researcher when consenting participants and deciding how to present and share their interpretations of the participants’ narratives. Questions have been raised regarding the ethical implications of imposing the researcher’s interpretation and meaning-making of the respondent’s narrative onto the respondent’s life story, and the possibility of this process altering participants’ perceptions of self [21].

Questions also exist in relation to the trustworthiness or validity of the interpretation in the stories [22]. Of particular concern to us, and the primary rationale for a number of researchers in using composite narratives to present their research findings, is that in focusing on small samples and particular experiences, it may be difficult to protect identities of the research participants, particularly when the cases are unique, sensitive, or involve insider research [8, 23, 24]. Protecting identities and assurances of confidentiality are central to gaining access to research participants, and these concerns have increasingly led researchers to explore the use of composite narratives in order to retain the benefits of narrative approaches whilst addressing some of their limitations. In medical education research, composite narratives could have an important role in presenting the findings of research involving sensitive subjects where identification might lead to concerns regarding career prospects, such as sexual harassment or assault [25, 26], and easily identifiable or minority groups, such as disabled students [27], and students from minority ethnic backgrounds [28], who might ordinarily be reluctant to participate in research. Understanding the experiences of these groups is vital in identifying areas of discrimination and in supporting groups who are victimised or harassed or who face previously unidentified challenges.

## Composite narratives

### What are composite narratives?

Narrative approaches to research have traditionally focussed on individual stories to allow for voices not usually heard to be prominent [15]. In contrast, a composite narrative synthesises the stories of research participants with the reflections and knowledge of the researcher to construct a storied account of the participants’ combined experiences whilst maintaining their anonymity. It conveys an appreciation of individual experience whilst identifying the shared or (in research where multiple composite narratives are constructed) divergent aspects of their stories.

Research utilising composite narratives comes from a range of different disciplines and explores experiences ranging from chronic illness to career transitions. Authors use a variety of terminologies to describe this approach. Tab. 1 outlines some of the key terms and definitions utilised within research from a range of disciplines; however, there are many others including: composite accounts [29], composite description/vignettes [30], composite personal meta-narrative [31], and embodied interpretation [32]. Several articles, in their fuller descriptions refer to hermeneutic phenomenology [18, 32, 33] and cite as influential the work of Todres [34] and/or Wertz et al. [18] to their approach [23, 30, 31, 33, 35]. What is common within this differing terminology is the emphasis on remaining authentic to the experiences of the individuals involved, the role of the researcher in interpreting that experience and, crucially, the desire to protect participants’ identities. We will focus our discussion on examples relevant to medical education, limiting our discussion of research from other disciplines to when a medical education example is not available.

**Table 1:** Composite narratives—terms and definitions

Term	Used by	Definitions
Composite fictions/stories	Piper and Sikes [24]	‘… we developed an ethnographic, composite fictional, storied approach … we fictionalised the accounts we were given—creating characters, contexts, and settings—inventing dialogue and crafting plots, but at the same time we did not make up ‘anything that directly related to peoples’ experiences and perceptions … we didn’t include everything because some events and occurrences were so singular that it would have been impossible to entirely disguise and anonymise them’ [36].’ [24, p. 568]
Composite first-person narrative (CFPN)	Wertz et al. [18]	‘The composite first-person narrative is a reflective story. It draws a composite picture of the phenomenon emerging from the informants. The composite is not a simple re-telling. It is interpretation by the researcher in several important ways: through her knowledge of the literature regarding the phenomenon under enquiry, through listening and hearing the stories told by the informants, and through her own reflexivity during the process … Use of the personal pronoun “I” is essential to the method. It indicates the composite-informant in the first-person sense as someone who typifies the general experience within a living and situated context.’ [18, p. 2–3]
	Biglino et al. [37]	‘The composite first-person narrative is a reflective story and results in a representation of the phenomenon amalgamating the voices of multiple participants … A composite approach incorporating narratives unearthed through formative research allows the researchers to use factually realistic details and shape a unified story’ [37, p. 3].
Composite life history/composite life story	Taber [38]	“I endeavoured to create a story that would highlight important aspects of each woman’s life as relates to the themes common to each,* thus combining paradigmatic processes focused on common experiences and narrative processes focused on individual stories*. This further connects the particular to the general, with an emphasis on social phenomena and organization” [38, p. 19].
Composite narratives	Willis [23]	“‘composite narratives’, in which a number of interviews are combined and presented as a story from a single individual” [23, p. 472].
	McElhinney & Kennedy [8]	‘Composite narratives … blend a number of accounts to convey an appreciation of individual experience whilst maintaining the anonymity of participants’ [8, p. 2].
	Creese et al. [11]	‘Composite narratives … involve the use of data from several interviews to tell a story framed as that of a single individual’ [11, p. 5].
	Johnston et al. [33]	‘Composite narratives are stories that are woven together to represent interview data from multiple participants, presenting complex ideas in a way that can impact on audiences and maximise reader resonance’ [33, p. 2].

### Why use composite narratives?

The central purpose of social research is to illuminate what has been previously hidden or unexplored in order to aid understanding and, in some cases, create change. Using composite narratives may increase the possibility of gaining access to hard-to-reach groups, such as senior politicians [23, 39], and experiences that are sensitive, such as teachers’ experiences of being accused of sexual abuse [24, 36]. A fundamental ethical principle in conducting research relates to the protection of participants, that they can be free to share their views and experiences without being identifiable [40]. This need to maintain anonymity can be seen as of central importance in medical education research. For example, for academic GPs working in a small teaching unit to discuss their experiences of becoming academics and feelings about the work, maintaining confidentiality, changing any identifiable details and using pseudonyms were key [8]. However, in the age of the Internet, the possibility of deductive disclosure [41] through random searches using snippets of data contained in research reports presents a serious threat to being able to guarantee this in certain circumstances [29]. Composite narratives afford researchers the ability to retain the benefits of narrative approaches whilst reducing the potential for exposing individual identities.

Whilst some approaches to the analysis and presentation of research, such as thematic analysis, can isolate the understanding of experience from its wider social milieu, composite narratives can situate experience within a context [18]. Some researchers have identified the value of composite narratives in demonstrating the commonalities of experience, for example in Wertz et al.’s composite narratives of adolescent girls’ experiences of obesity and women experiencing distress in the menopause transition [18], whilst others have developed several composites to demonstrate the differences in experiences, such as GPs pathways into academic medicine [8]. Composite narratives have been used in research where the participants have in common their core experience, for example academic GPs [8], and to explore the different relationships to an event relating to contrasting positions, for example, the experiences of mothers and midwives relating to the removal of babies at birth [42, 43].

A further proposed reason for using composite narratives is to increase a reader’s engagement with the lives and experiences of research participants [11, 43]. Taber [38, p. 18–19] has argued that presenting participant stories in long-form is important to move beyond disparate themes and issues and understand the complexities of people’s lives, ‘to be pulled into a narrative, a deepening engagement with individual lives that have common threads’. Composite narratives allow for presentation of these complexities without linking them to individual participants. The presentation of research as narratives also increases the potential for research to be accessible to a wider audience, for use in teaching, policy and community contexts outside academia [23]. This accessibility to a lay audience has been shown to provide opportunities for behaviour change when stories, fictionalised, authentic or composite, are used in public health campaigns, for example on hypertension and smoking cessation [44].

## Process approaches

### Methodological background

The construction of composite narratives as a research output has been employed by researchers using a variety of qualitative approaches and methodologies (including phenomenology, ethnography, narrative research and grounded theory) and a range of methods of data collection such as interviews [8, 23, 36, 39], written biographies and autoethnography [8], creative writing and artwork [37], photographs and field notes [42, 43], and audio recordings of workshops [37]. Composite narratives can form one part of the outputs of a study [11, 18] or be the main focus of the data [8, 37, 38].

Composite narratives can be used either as a methodological approach integral to the research design, analysis and outputs [8, 37], or as a vehicle for presentation of research outputs which may be decided on after completion of the initial analysis [11, 18, 23, 39]. The methodological approach and philosophical underpinnings of the research influence the form of the composite narrative constructed from the data. Most research employing composite narratives is from a constructivist philosophical standpoint, subscribing to the belief that all knowledge is subjective and socially constructed [40]. This is important because such a philosophical stance acknowledges the importance of the researcher’s interpretation in the research process and therefore accommodates more involvement of the researcher’s voice in the findings than a realist or positivist approach would allow**—**such approaches requiring the bracketing or separation of the researcher’s influence on the results.

Examples from the literature of ways in which composite narratives are constructed include first-person narratives [8, 18, 37, 38, 42, 43], third-person narratives [11, 23, 39], and ‘composite fictions’ [24]. Tab. 2 summarises examples of differences in the methodological approach, method of data collection and type of composite narrative produced from a few key studies. The table draws on examples from health and social sciences research, as examples from the medical education literature are limited.

**Table 2:** Methodological approaches to constructing composite narratives

Methodological approach	Examples	Data collection method (Participant numbers)	Features of composite narrative(s)
Ethnography	Piper and Sikes [24]	Narrative interviews Written narratives (*n* = 25+)	‘Composite fictions’ First-person account with characters, settings, context, and plots created to protect identities of participants
Phenomenology	Biglino et al. [37]	Creative writing, drawing, audio recordings of workshop (*n* = 5)	First-person narrative Three research group members each produced a composite narrative, these were combined to produce a single final narrative
	Wertz et al. [18]	Interviews Nosek (*n* = 19) Wertz (*n* = 15) Marlow (*n* = 17) McNeish (*n* = 15)	First-person narratives Aim to convey both structure (narratives illustrate the main themes) and texture (richness of participants’ experiences)
Narrative research	McElhinney and Kennedy [8]	Interviews, Biographical written narrative, Autoethnography (*n* = 9)	First-person narratives Combined narrative data from participants with autoethnography, narrative arcs, phraseology and style of individual narratives incorporated
	Willis [39]	Interviews (*n* = 14)	Third-person narratives Descriptions of interviews with composite characters. Direct quotations from transcripts incorporated into narratives
	Taber [38]	Interviews (life history research) (*n* = 3)	First-person narrative Combined text from all interview transcripts to create a composite
Qualitative framework analysis	Creese et al. [11]	Interviews (*n* = 48)	Third-person narratives Scaffolded by theoretical framework
Grounded theory	Johnston, Wildy & Shand [33]	Interviews (*n* = 25)	First-person narratives 24 narratives produced, one to illustrate each of the findings

Researchers in the phenomenological tradition use composite narratives as a way of conveying both the structure (the themes characterising the phenomenon) and the texture (the richness of the experiences of the participants) of the research findings [18, 34]. This is in keeping with the aim of phenomenological research to describe and explain the subjective lived experiences of individuals [40], and its emphasis on the importance of the first-person viewpoint in conveying this richness of experience. In healthcare research, phenomenological researchers argue that composite narratives are an accessible means of conveying research findings which can enrich healthcare practitioners’ understanding of their patients’ lived experiences and facilitate empathic care [18, 37].

Narrative research sets out to explicitly gather data with an emphasis on the story of an aspect of an individual’s life and is a natural environment for the evolution of the composite narrative as a research output [8, 23, 38, 39, 42, 43]. Narrative research acknowledges that the data gathered illuminates both the participants’ experience and their reflections on the experience as they frame it for telling, choosing language, emphasis and shared reflections. Composite narratives produced through narrative research include both first [8, 38, 42] and third [39] person composites. In our own research, we used data in the form of written narratives, interviews and an autoethnographic study to produce composite narratives describing the experiences of academic GPs [8]. Autoethnographic study was included as one of the authors was an insider researcher and this informed the choice of the first-person voice, emphasising the intertwining of the researcher’s voice with those of the other participants in creating the narratives from their shared experiences. This research is an example of the composite narrative being integral to the design, analysis and outputs of the research; the decision to create composite narratives was embedded in the research design and specified when consenting participants. Written narratives were first analysed individually taking a thematic narrative approach [5], with similarities of the narrative arcs and shared experiences between groups of participants identified. The authors then started drafting composite narratives for each of these groups, highlighting these commonalities in the narratives. This initial stage of the analysis helped to shape the focus for the subsequent interviews which explored the similarities and differences between the participants in more depth. The texts from the transcribed interviews were then also analysed individually, before being synthesised with the three developing composites. Taber [38] and Marsh et al. [42] also created first-person narratives to present their research findings. In both these studies, the authors created their narratives by blending the words of all participants to create a composite.

A different approach was taken by Willis [39], in her research exploring how senior politicians understand their role in relation to climate change. Following the initial analysis and coding of her data using NVivo software, she found that ‘The overall picture thrown out by the interview data was both more tangled and richer than anticipated’ with a ‘complex web’ of ‘individual motivations and outlooks’ [p. 481]. Her decision to use composite narratives was taken as a means of addressing the challenge of conveying ‘the richness and complexity of individual accounts’ while honouring the ‘need to ensure anonymity’ [p. 481]. She explains that she chose to use composite narratives to protect the identities of her participants and to explore context and complexity rather than defining differences and categories [23]. Willis developed four composite narratives presented as reports of interviews with a composite character, using quotations derived from the interview transcripts.

In their ethnographic study exploring the experiences of teachers wrongly accused of sexual abuse of pupils, Piper and Sikes describe the creation of ‘composite fictions’ which they describe as ‘bring[ing] the written product of social research closer to the richness and complexity of the lived experience’ ([45, p. 7–8], cited in [24, p. 568]). Given the sensitivity of their research subject, they needed to convey the experiences of their participants while avoiding any potential for identification from any details presented in the research findings. They addressed this by creating ‘composite fictions’ where they created settings, context and timelines while ensuring they did not invent the experiences and perceptions of participants conveyed in the narratives ([36, p. 43–44], cited in [24, p. 568]).

More recently a large qualitative study exploring the wellbeing of doctors in Ireland during the COVID-19 pandemic [11] produced composite narratives that summarised their key findings as third-person accounts, scaffolded by the theoretical model they used to analyse their large body of qualitative data.

### Presentation

As composite narratives tend to be lengthy pieces of writing, the presentation may be adapted depending on the purpose or intended form of publication. They may be presented within research papers in their entirety [11, 18, 24, 37] or as excerpts from the narrative [8] with the complete narratives appended as supplementary materials, a development made possible with online publishing. A recent Scottish study exploring the experiences of doctors during the COVID-19 pandemic [10] has led to the production of animations portraying composite narratives, aiming to enhance doctors’ wellbeing by encouraging them to seek support by disseminating their findings in an accessible format.

### Establishing trustworthiness

As Polkinghorne [46, p. 471] notes, ‘attention to the judgments about the validity of research—generated knowledge claims is integral to all social science research’. Some readers of research, particularly those working in a positivist or post-positivist research paradigm, may question the validity of research that involves the ‘storying’ or ‘re-storying’ of data. Such apprehension about the trustworthiness of the interpretation may be exacerbated where stories are combined and/or joined by the voice of the researcher. A core value of research is that its findings are accepted and that it holds the possibility to influence change [22]. In order to uphold these values, narrative (and composite) research must demonstrate ‘the procedures used to ensure that its methods are reliable and that its findings are valid’ ([47], cited in [22, p. 12]). For researchers working within a constructivist paradigm, however, trustworthiness and the extent to which the researchers or relevant communities can feel safe in acting upon the findings [48] are held to be more helpful quality criteria than validity.

Attention to the elements of trustworthiness (credibility, transferability and dependability) as set out by Lincoln and Guba [49] are important in establishing the value of qualitative research methods. This is particularly true for methods that are novel within a particular field. With this in mind, medical education researchers creating composite narratives should consider steps such as prolonged engagement with their subjects, triangulation of data, peer debriefing, archiving of raw data for future comparisons and member checking as vital components in establishing credibility [49]. Transferability in the constructivist context is not directly comparable with the external validity expected in the positivist paradigm. However, the provision of detailed description and contextualisation will allow readers to make informed decisions about the appropriateness of transferring the themes of the findings and acting upon them in their own context. Finally, dependability can be strengthened by having multiple researchers involved in the design, analysis and outputs of the research. Establishing the trustworthiness of research findings presented as composite narratives therefore requires clear description of the research design including the steps taken to establish credibility and of the approach taken to transform individual narratives into composites [50]. Composites must include thick description and context in order for readers to make decisions about transferability of the findings.

Reflexivity is also vital in ensuring the trustworthiness of this method and the researcher’s role should be acknowledged and accounted for. In some methodological approaches, such as interpretative phenomenology and those that combine autoethnography with other participant data, reflexivity is explicit in the method. In interpretive phenomenological research, the interpretation by the researcher of the phenomenon described is a vital aspect of the research [18] and the composite conveys both the phenomenon and the researcher’s interpretation of its meaning. Similarly, combining autoethnography with data from other participants makes explicit the author’s entwinement with the subject [8]. Willis [23] acknowledges her involvement by situating herself in the narratives as the interviewer narrating her third-person accounts of the interviews and including her observations on the composite characters described.

## Conclusions and implications for medical education research

Composite narratives have recently started to be used in medical education research, both as a component of the methodological approach [8], and as a means of presenting findings [10, 11]. Their use can be valuable in protecting the identities of participants in small or easily identifiable groups, and in conveying research findings in an accessible format. Composite narratives have the potential to present research findings as holistic, contextualised, and engaging accounts, accessible to non-specialist audiences and able to convey emotionally authentic descriptions of participants’ experiences to the reader. They can convey temporal sequence and be used to illustrate both commonalities of experience between participants, and the contrasting experiences of different participants in a group, depending on the number of composites produced.

In medical education research, composite narratives are valuable in disseminating findings to those out with the field but with key interests in shaping policy, for example, by illustrating the multiple challenges faced by doctors in training in a pandemic in a way that isolated quotes might not [11]. The potential for composite narratives to highlight the challenges faced by underrepresented or marginalised groups within medical education, through enabling such voices to be heard without being identifiable as a single individual, could be a powerful means of advocating for change and addressing discrimination and inequalities.

As we have outlined earlier in this paper, narrative can be used in research in a multitude of ways: as a methodological approach, a means of data collection, and as a way of representing research findings. The creation of composite narratives can be employed in the analysis of the data [8, 37] as well as in the presentation of the research findings of research from multiple epistemological positions [8, 11, 18, 24, 33, 37, 39]. These positions, and their attendant conceptualisations of narratives, will influence the role of composite narratives in the research. Researchers employing composite narratives should be clear that their conceptualisations of narrative are consistent with their epistemological position, and that they address questions of trustworthiness within their methodological approach and interpretation of the findings in a way that is consistent with their epistemological assumptions.

Researchers using composite narratives should be clear about their reasons for choosing this approach and ensure transparency and rigour in their use of the method to ensure trustworthiness. It should be clear whether the production of composite narratives was integral to the research design and whether the intention to present findings as composite narratives was explicit to research participants at the outset. Transparency is also vital as to whether the construction of narratives was intrinsic to the analytic process or decided on after analysis was complete as a means of disseminating data. If the production of composite narratives was decided on as a means of presenting findings after analysis of the data, researchers should make it clear why this approach was chosen, particularly if chosen to address challenges such as the presentation of sensitive or personally identifiable data. Researchers should clearly describe their approach to construction of their composite narratives, making it clear to readers how the data has informed the narratives, to counter criticisms of the narratives being so far removed from the data as to be untrustworthy.

A potential criticism of the method is the role of the researcher in the creation of the narratives and the threat to trustworthiness that this could pose. For this reason, researchers should be clear about their own positioning and role in the narrative production, and ensure reflexivity by acknowledging and accounting for their role and background. Where reflexivity is not explicit in the methodological approach, such as when researchers have decided to create composites from a body of data they have collected to increase accessibility or to tackle a problem such as ensuring anonymity, researchers should take care to describe their involvement in the research and narrative construction and ensure reflexivity is incorporated.

Finally, the accessibility of findings presented as composite narratives makes member checking a more realistic proposition as the blending of accounts from multiple participants could mitigate some of the ethical concerns raised regarding the ownership of the narratives and the imposition of the researcher’s interpretation [21]. Member checking should therefore be incorporated wherever possible in order to increase trustworthiness.

These considerations of epistemological and methodological coherence, trustworthiness, and reflexivity will be vital in the development of the composite narrative as an accepted methodological approach in medical education research. As this is very much a developing approach within the field, the authors advocate a considered approach to employing composite narratives in research design, in order to better develop the trustworthiness and acceptability of this novel narrative research method in medical education research.
